# Celebrating 30 years of the international network of agencies for health technology assessment (INAHTA)

**DOI:** 10.1017/S0266462325000042

**Published:** 2025-02-03

**Authors:** Tracy Merlin, Sophie Söderholm Werkö, Alric Rüther, Tara Schuller, David Hailey

**Affiliations:** 1 Adelaide Health Technology Assessment (AHTA), School of Public Health, University of Adelaide, SA, Australia; 2 The Swedish Agency for Health Technology Assessment and Assessment of Social Services (SBU), Stockholm, Sweden; 3 Institute for Quality and Efficiency in Health Care / Institut für Qualität und Wirtschaftlichkeit im Gesundheitswesen (IQWIG), Cologne, Germany; 4 Institute for Health Economics (IHE), Edmonton, Alberta, Canada; 5 School of Information Systems and Technology, University of Wollongong, New South Wales, Australia

**Keywords:** Collaboration, Network, International, Health technology assessment, Sustainability

## Abstract

It is not often that international collaborations are sustained for any significant period, let alone for three decades. However, despite relying on largely voluntary contributions of individuals within its member institutions, the International Network of Agencies for Health Technology Assessment (INAHTA) has not only been an example of sustained collaboration over 30 years but also an example of how an initially modest collaboration can grow and thrive. Current and former serving Chairs and secretariat of the Network have come together to review network documents and outputs and reflect on the history of INAHTA, since its inception in Paris in 1993. Building on the paper from Hailey et al 2009 that documented the growth of the network after 15 years, we have considered and documented the factors that we believe have helped sustain the network and enable it to flourish in the subsequent 15 years. We have also considered the various challenges experienced along the way, as these too can aid in making a collaboration stronger. Future directions for the network have also been contemplated, given the evolving nature of HTA and the regional collaborations that have recently emerged. We hope that by sharing the lessons learned from this living example of international global collaboration relationships between like-minded organizations can be similarly fostered and enhanced into sustainable collaborations, for the benefit of all.

## Introduction

In English custom, a pearl is the symbol for a 30-year wedding anniversary. In 2023 the International Network of Agencies for Health Technology Assessment (INAHTA) reached the pearl anniversary of its formation in Paris in 1993. This anniversary was a significant milestone in the network’s history and so at the 2023 INAHTA Congress in Adelaide, Australia, it was agreed that it was timely to review the achievements, challenges, and future directions of this unique organization (1). This review was considered particularly relevant given the current global interest in networks and collaborations in HTA, with the topic of the HTAi Global Policy Forum in January 2024 being *Designing collaborations involving HTA: Finding the rhythm for success.*

Four of the coauthors of this paper are current (AR) or former (TM, SW, DH) Chairs of INAHTA, with an intimate knowledge of governance of the network. They represent a combined 28 years on the Board of INAHTA. One of the coauthors (TS) has been responsible for managing the INAHTA secretariat for over 15 years and one of the coauthors (DH) was present during the formation of the network. This in-depth knowledge has been supplemented with reviews of INAHTA Annual Reports, membership lists, Board minutes, and correspondence to provide an overall picture of the operation of the network over 30 years.

A seminal publication on the history of INAHTA was written in 2009 by one of our coauthors (DH) (2). We have used this paper as the baseline for determining what has happened with the network in the intervening 15 years. As the only *global* network of not-for-profit agencies that assess health technologies in support of regional or national governments, INAHTA is a microcosm for the development of HTA globally. It has many lessons to offer about how an international network can grow and thrive.

## Formation of INAHTA: “From Formation to Fruition”

INAHTA was established in 1993 at a meeting in Paris, reflecting a need for better communication and collaboration between HTA agencies (3). This followed discussions between HTA agency representatives at meetings of the International Society of Technology Assessment in Health Care (ISTAHC). Further discussions on the structure and activities of INAHTA took place in 1994 and a modest secretariat was established at the Canadian Coordinating Office for Health Technology Assessment (CCOHTA) in Canada. In 1996, the secretariat moved to the Swedish Agency for Health Technology Assessment (SBU) and staff there managed the network until 2012, supporting the development of many activities. After a short period at the German Institute for Medical Documentation and Information (DIMDI), and then at the Australian Safety and Efficacy Register of New Interventional Procedures – Surgical (ASERNIP-S) in Australia, together with SBU, in 2013 it then returned to Canada but this time to the Institute of Health Economics (IHE). IHE continues to provide INAHTA secretariat services today.

Rules for full membership in the network are essentially unchanged from its establishment, covering not-for-profit status, the requirement for an HTA function and relationship to government, and the public sources of funding. The provision of free access to publicly available reports to other INAHTA members was an additional requirement.

## Growth and development of the network

**Figure 1.**
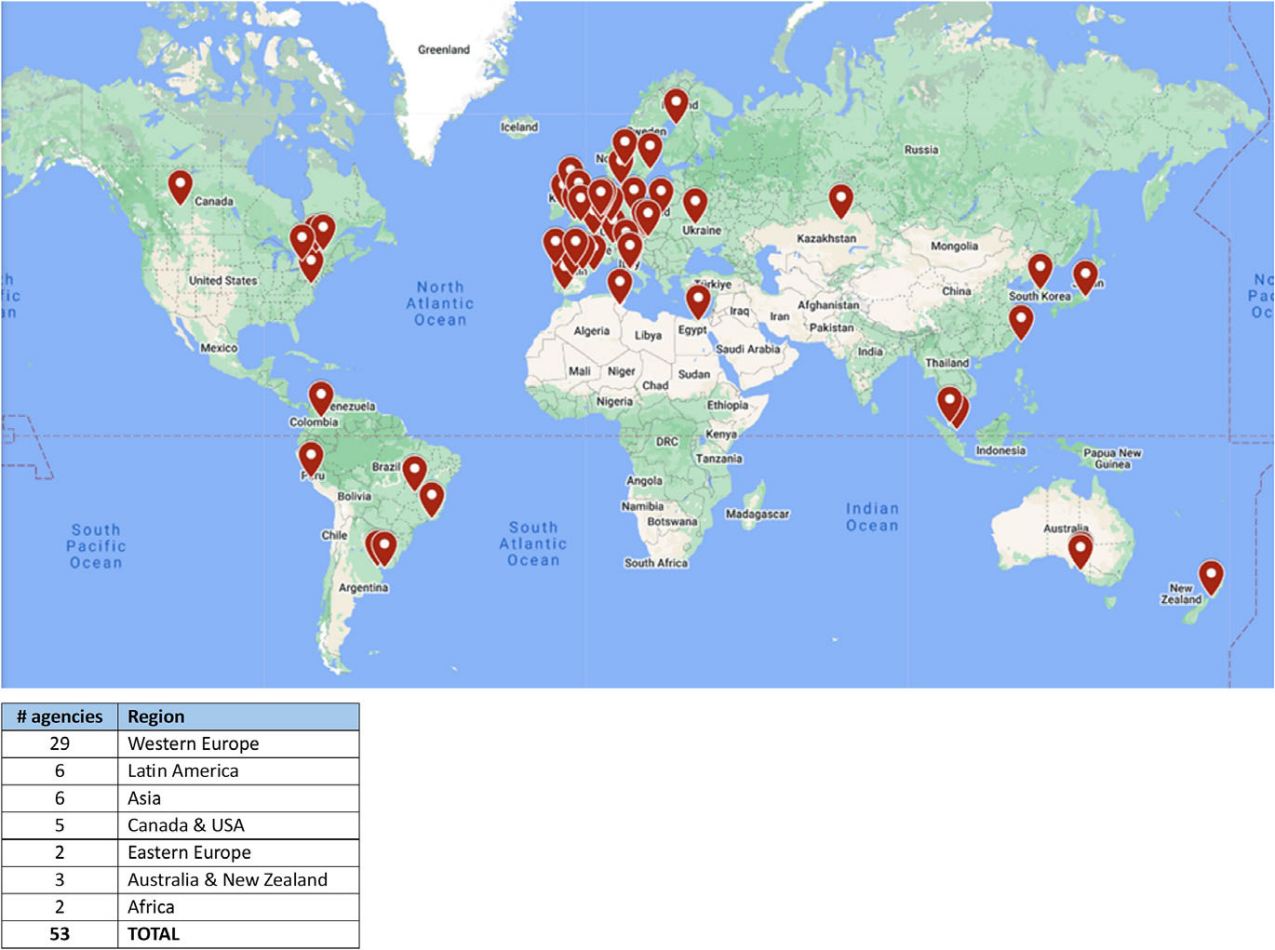
Map of INAHTA members as at November 2024 (53 agencies in 34 countries).

INAHTA was founded by the heads of 13 HTA agencies in nine countries (Australia, Canada, France, the Netherlands, Spain, Sweden, Switzerland, the United Kingdom, and the United States) who recognized the value of building connections and opportunities to exchange information among the public HTA agencies. The original founding members of INAHTA came from high-income countries in Europe, North America, and Australia, but over the years, the INAHTA membership has expanded immensely (Figure 1). As of November 2024, INAHTA has fifty-three member agencies from thirty-four countries: twenty-nine from western Europe, six from Latin America, six from Asia, five from Canada and the United States, three from Australia and New Zealand, and two from Eastern Europe, as well as two agencies from Africa. INAHTA membership has also diversified over the years, and in 2023, 20 percent of member agencies were from low- and middle-income countries. Table 1 presents a comparison of global membership in 1993, 2009, and 2023.

**Table 1.** INAHTA members: number of agencies by country, 1993, 2009, and 2023

Country	1993	2009	2023
Argentina		1	1
Australia	1	3	2
Austria		1	2
Belgium		1	1
Brazil		1	2
Canada	1	4	4
Chile		1	
Colombia			1
Denmark		2	1
Egypt			1
Finland		1	1
France	2	2	2
Germany		2	2
Israel		1	
Ireland			1
Italy		1	3
Japan			1
Kazakhstan			1
Korea		1	1
Lithuania		1	
Mexico		1	
Malaysia		1	1
New Zealand		1	1
Norway		1	1
Peru			2
Poland		1	1
Singapore			1
Slovak Republic			1
Spain	2	6	6
Sweden	1	1	1
Switzerland	1	1	1
Taiwan		1	1
The Netherlands	3	3	2
Tunisia			1
UK	1	4	4
Ukraine			1
Uruguay			1
USA	2	2	1
Total	14	46	54*

* As of November 2024 there were fifty-three agencies (see Figure 1).

However, growing the Network has not been a primary focus of INAHTA’s strategic planning. Rather, it is the quality of the collaborations and the networking that has been of prime importance. The initial rapid growth of the Network and then settling at a fairly consistent ‘steady state’ has been ideal because it has allowed strong relationships to form between the members. Since its inception, INAHTA has demonstrated success in improving the exchange of information and the level of collaboration and cooperation among member agencies in undertaking HTA-related projects. Organizational arrangements for the interaction of INAHTA agencies with the decision makers they inform have generally been well established. However, these are often subject to change, and so sometimes members have had to leave the Network because of changes in remit, closure, or restructure, reflecting national political and governance decisions.

## Activities of the network

Apart from the specific network governance activities such as the Annual Business Meetings, INAHTA offers their members a range of activities today.

The structuring and organization of INAHTA’s work have changed from the early working groups in the areas of internal and external communication, education & training, HTA Impact, and HTA Quality Assurance to today also encompassing, besides the *Board standing committees* (e.g., External partnership committee, Nominations committee, Congress Committee, HTA Database Steering Committee, Scientific Program Committee), *Task Groups* and *Learning Groups.* All groups are made up of volunteers from member agencies. *Task groups* are created for a specific task and once the task has been accomplished, the group is dissolved, for example, the Membership Criteria Task Group whose task it was to review the membership criteria of the network. *Learning groups*, on the other hand, have no other task than to share knowledge and experiences between members. There are currently four Learning Groups: Patient Engagement, Real World Evidence, Environmental Sustainability, and Qualitative Evidence. The activities offered in each Learning Group will differ according to the wishes of the group members, but most offer regular webinars, occasionally with invited external speakers. The Learning Groups act as both learning and sharing opportunities and have been very popular with members. The formation of a Learning Group on a topic of interest occurs at the instigation of the members. In addition to the Learning groups, INAHTA has a Scientific Program Committee whose task is to put together INAHTA’s *Scientific Program*, which is delivered virtually and all year round.

The *Listserv* is a service for members to ask any questions of other INAHTA member agencies, usually on specific assessments. This is a quick and easy way for the members to share information that can help them with their own assessment activities. The questioner receives the responses immediately and all responses are summarized and shared with the members yearly.

Information on practice and opinions from member agencies are from time to time collected through surveys within INAHTA. These have included surveys and published papers on “Hot topics” and the top ten challenges experienced by members (4), member agencies’ experiences with patient engagement (5), and the methods INAHTA members use to conduct their HTAs (6).

*The International HTA Database* is another service, owned and provided by INAHTA to members and to the HTA community, as a whole, and it is freely and publicly accessible. The Database is where information on assessments by members is published, including their planned, ongoing, or recent HTA reports. Members of INAHTA no longer produce INAHTA Briefs (3), but the use of the Database has expanded. There is also a possibility for groups of members to have their own restricted space in the Database where they can share this type of information with each other (e.g., in another language or within specific regional boundaries). The international *HTA Glossary* is another service that is publicly provided by INAHTA in collaboration with HTAi, with members providing in-kind secretarial support for the different language editorial boards – the English Editorial Board providing definitions of HTA-related terms and other Editorial Boards translating these terms into French, German, Spanish and Russian.

Other tools that INAHTA has produced, and which are provided to members, have included an impact framework (7), reference documents that provide guidance on various aspects of HTA, a checklist for HTA reports, and most recently a member manual.

The major annual activity is the INAHTA *Congress*, which is the network’s single face-to-face (now hybrid) meeting for all members, which normally lasts 1.5 days, starting straight after the end of Health Technology Assessment international (HTAi)‘s annual meeting. INAHTA’s external partners are usually invited to the first day of the Congress and the focus of these days is on scientific, methodological, or other HTA-related topics from the agency’s perspective. The Congress is unique in that it is the only place, globally, where HTA agencies can meet on their own. This is where ideas are presented, formulated, and sometimes decided upon.

It was for example at the INAHTA Congress in 2019, that members decided INAHTA should start to produce *Position Statements.* These are general declarations that mark a particular point of view or standpoint supported or approved by 70 percent or more of INAHTA members. They can focus on general issues, methods, definitions, or processes relevant to HTA and to HTA agencies. They are however not intended to deal with specific interventions or health technologies. To date, INAHTA has published three position statements (the uniqueness of INAHTA (1), patient involvement (8), and disruptive technologies (9)). At the Annual Business meeting in 2023, members decided, based on a recommendation from the Position Statement Pilot Process Evaluation Task Group and the Board, to make position statements permanent products of INAHTA.

Early in the life of the network, joint projects between INAHTA agencies on the assessment of specific technologies were conducted (2), but this is no longer the case. This was partly due to the creation of regional networks, that is EUnetHTA, HTAsiaLink, and RedETSA, whose members are closer in alignment on approaches to HTA than across the whole network.

## Interaction with other organizations

Public HTA agencies must be informed early about the latest developments in HTA and must have the opportunity to collaborate with relevant expert committees and organizations. Furthermore, HTA, as a multidisciplinary field, requires the integration of various disciplines, where external expertise is highly beneficial. This applies not only to current projects but also to capacity building, the establishment, and development of HTA organizations and their staff. Interaction with international organizations, outside the network, plays a significant role in these efforts.

From its inception, INAHTA recognized the importance of exchanging knowledge with external organizations. Initial contacts have since evolved into trusted relationships. A prime example is Health Technology Assessment International (HTAi), the international society for HTA. Both INAHTA and HTAi, for example, send observers to each other’s board meetings and collaborate on numerous projects, such as the *HTA Glossary.* The recent renewal of the Memorandum of Understanding (MoU) expanded the collaboration between the working groups of both organizations, organized through a joint work plan.

INAHTA also maintains a long-standing partnership with the World Health Organization (WHO), focusing on capacity building, particularly in countries with emerging HTA structures – a very successful collaboration that will continue to grow in the coming years.

Another critical area is the exchange with regional HTA networks. INAHTA is closely connected with the HTA Network for Asia and Oceania (HTAsiaLink) and the HTA Network for Latin America (RedETSA). In Europe, INAHTA has supported the EUnetHTA project series from the beginning of the establishment of the EU-HTA Regulation. This collaboration will likely continue with the EU-HTA Coordination Group and the Heads of Agencies Group (HAG).

Additionally, INAHTA maintains connections with related fields such as clinical practice guidelines development (Guidelines International Network (GIN)) and horizon scanning (International Health Tech Scan (IHTS)). Over the first 30 years of INAHTA, it has become evident that exchange and collaboration with international organizations in HTA are extremely valuable and enriching for the network, as they provide a mechanism to learn from each other. INAHTA will continue to nurture and expand these relationships over the next 30 years.

## Challenges

INAHTA has also had its share of challenges since it was established. These can be categorized into four key areas: (i) governance, (ii) membership, (iii) collaboration, and (iv) scale and focus. *Governance issues* that have arisen include whether to become a legal entity and formally register as a not-for-profit organization. This has been discussed many times by the INAHTA Board, with advantages and disadvantages canvassed regarding the decision. The advantages mainly relate to improving INAHTA’s ability to directly enter contracts, rather than doing this through the HTA agency that provides the network secretariat. The disadvantages of becoming a legal entity related to the fact that most of INAHTA’s members are government agencies and so may not be able to become a member of another formal organization. Other governance issues that have proved challenging at times have included getting members involved in establishing and maintaining the network’s bylaws and ensuring the strategic directions of the network are appealing to a range of different member agencies that are at different stages of maturity. COVID-19 brought challenges with the need to host a virtual Congress and annual business meeting at short notice; it was also an opportunity, however, as it was found that online attendance allowed much broader participation from members and so a hybrid format of the Congress, online annual business meeting, and the year-round online scientific program was instituted from 2022 onwards.

INAHTA has clear *membership criteria* (10) and decisions regarding whether to admit (or not) new members to INAHTA are usually uncontroversial. However, over the years a handful of applications have raised challenges, mostly as a consequence of political disagreements between the governments of existing members and those of applicant agencies. In such cases, the Board reflected on INAHTA’s values of collaboration, communication, continuous learning, independence, trust, and transparency. If these values were unlikely to be achieved between the new applicant and existing members (noting many of our members are governments), then membership was not granted.

Collaboration with external partners is very important to INAHTA but this can occasionally lead to tensions when communication on joint activities has not been ideal or when joint activities have not been equally supported or expedited. Another challenge, this time related to internal collaboration among members, is the development of INAHTA Position Statements. As described earlier, these are an important initiative from the network to help define who INAHTA is and what it stands for. However, gaining consensus (defined as 70 percent agreement among member agencies) can be difficult when the topic is controversial or when it is a newly emerging area with divergent perspectives. Fortunately, the shared purpose of INAHTA and the mutually respectful relationships of its members have allowed disagreements to be resolved or accepted and understood. INAHTA today has agreed on and published three position statements (1;8;9).

INAHTA’s final main challenge has been about finding a focus to build scale. INAHTA’s modest resourcing comes from member subscriptions and the secretariat has traditionally been very small and primarily focused on supporting networking opportunities among members. However, as INAHTA’s membership has grown and diversified, the activities of the network have adapted to become more responsive to the pressing issues that members face in producing high-quality HTA for decision makers in constantly evolving health systems. Although regular strategic planning has been a part of INAHTA’s activities for many years, understanding the changing needs of members remains an ongoing challenge. The Board has in turn stepped up the intensity of monitoring the network’s strategic goals to more effectively link these to the operational planning of secretariat resources. The greater operational focus has now allowed better directed resourcing and support, including increasing the size of the secretariat, and sourcing additional ad hoc support when required, to assist with priority activities, including the development of new Learning Groups, a year-round virtual scientific program, and responsibility for the international HTA database (hosted by INAHTA from July 2020, although contributed to since its inception).

## Concluding comments

This exploration of the past 30 years of INAHTA has highlighted the importance of maintaining close communication with members, identifying their needs and wants and prioritizing these in strategic and operational plans, and having regular in-person and online scientific meetings as well as social events to foster relationships and opportunities for informal advice and learning. Members of the network have a common purpose, even though there is a great deal of variation in how HTA is applied due to health system differences. This common purpose and INAHTA’s values, which guide the governance of the network on a daily basis, have been instrumental in sustaining the network for 30 years.

INAHTA was created to reduce an “irritation” around unwarranted duplication of effort in HTA globally. As Hailey stated in 2009, “There was increasing perception that cooperation between agencies would reduce unnecessary duplication of activities, enable a more efficient sharing of expertise and information, and advance the field of HTA” (2). When celebrating INAHTA’s pearl anniversary, members learned that pearls are formed when an irritant in an oyster shell is covered by layers of nacre or mother of pearl in order to isolate and neutralize it. This is symbolic of the growth of INAHTA: from something that was small and irritating, something precious has been created.
